# Patient Experiences of Web-Based Cognitive Behavioral Therapy for Heart Failure and Depression: Qualitative Study

**DOI:** 10.2196/10302

**Published:** 2018-09-05

**Authors:** Johan Lundgren, Peter Johansson, Tiny Jaarsma, Gerhard Andersson, Anita Kärner Köhler

**Affiliations:** 1 Division of Nursing Science Department of Social and Welfare Studies Linköping University Norrköping Sweden; 2 Mary Mackillop Institute Australian Catholic University Melbourne Australia; 3 Division of Psychology Department of Behavioural Sciences and Learning Linköping University Linköping Sweden; 4 Department of Clinical Neuroscience Karolinska Institute Stockholm Sweden

**Keywords:** cognitive therapy, content analysis, depression, heart failure, internet, patient experience, telehealth

## Abstract

**Background:**

Web-based cognitive behavioral therapy (wCBT) has been proposed as a possible treatment for patients with heart failure and depressive symptoms. Depressive symptoms are common in patients with heart failure and such symptoms are known to significantly worsen their health. Although there are promising results on the effect of wCBT, there is a knowledge gap regarding how persons with chronic heart failure and depressive symptoms experience wCBT.

**Objective:**

The aim of this study was to explore and describe the experiences of participating and receiving health care through a wCBT intervention among persons with heart failure and depressive symptoms.

**Methods:**

In this qualitative, inductive, exploratory, and descriptive study, participants with experiences of a wCBT program were interviewed. The participants were included through purposeful sampling among participants previously included in a quantitative study on wCBT. Overall, 13 participants consented to take part in this study and were interviewed via telephone using an interview guide. Verbatim transcripts from the interviews were qualitatively analyzed following the recommendations discussed by Patton in *Qualitative Research & Evaluation Methods: Integrating Theory and Practice*. After coding each interview, codes were formed into categories.

**Results:**

Overall, six categories were identified during the analysis process. They were as follows: “Something other than usual health care,” “Relevance and recognition,” “Flexible, understandable, and safe,” “Technical problems,” “Improvements by real-time contact,” and “Managing my life better.” One central and common pattern in the findings was that participants experienced the wCBT program as something they did themselves and many participants described the program as a form of self-care.

**Conclusions:**

Persons with heart failure and depressive symptoms described wCBT as challenging. This was due to participants balancing the urge for real-time contact with perceived anonymity and not postponing the work with the program. wCBT appears to be a valuable tool for managing depressive symptoms.

## Introduction

Approximately 20% of the heart failure population suffers from depressive symptoms [1,2]. Depressive symptoms in heart failure are associated with a poorer health-related quality of life [3,4], morbidity [5,6], and increased mortality [1,2,7]. Depression is also associated with impaired self-care ability [8-11]. Self-care can be complex for persons with heart failure and requires knowledge (about heart failure), decision making, and practical skills [12]. The complexity of self-care may contribute to the poor prognosis and outcomes among persons living with heart failure [13,14]. Additionally, when depressive symptoms coexist with heart failure, the situation may be even more problematic because depression can impede learning ability, decision making, and task performance [15,16].

Guidelines on heart failure from the European Society of Cardiology (ESC) [17] and the American College of Cardiology/American Heart Association (ACC/AHA) [18] point out depression as a common comorbidity in heart failure resulting in poorer prognosis and reduced health-related quality of life. The ACC/AHA guidelines state that the current evidence is too weak to give recommendations on treatment of comorbid depression [18]. According to ESC guidelines, routine screening for depression in heart failure is good practice, and psychosocial and pharmacological interventions are regarded to be helpful [17]. However, no clear recommendation regarding the management or treatment of depression in heart failure is provided [17,18]. Generally, depression is effectively treated with pharmacological and psychotherapeutic interventions [19]. However, treatment of depression in patients with heart failure is challenging. Tricyclic antidepressants should be avoided because of their negative effects on the heart [17] and selective serotonin reuptake inhibitors have not shown greater effect on depressive symptoms than placebo [20]. Another challenge is that psychotherapeutic competence in the health care system is lacking [21]. To sum up, a large group of individuals do not receive adequate treatment while living with heart failure and depressive symptoms owing to lack of clear recommendations and psychotherapeutic competence. Cognitive behavioral therapy (CBT) for depressive symptoms in persons with heart failure has shown promising results in reducing depressive symptoms in a few studies [22,23] and in secondary prevention in persons with coronary heart disease [24]. The effect of CBT on self-care was similar compared with controls without feedback [25] or usual care [22]. Face-to face CBT is resource demanding because it requires health care personnel allocated to support individual patients for approximately 1-2 hours per week over 10-20 weeks [26]. Thus, owing to a lack of trained CBT therapists and time in the health care system, other ways to provide CBT have been suggested [21]. One such form of CBT is Web-based CBT (wCBT). wCBT is an effective treatment for mild to moderate depression; wCBT has thus been suggested as a treatment option for depressive symptoms in patients with chronic somatic diseases, such as heart failure [27]. wCBT programs need to be adapted to fit the context of the specific somatic disease [28-30], and such programs for depressive symptoms in persons with heart failure are still rather new and unexplored [31].

At a conceptual level, wCBT can be described as a form of telehealth, that is, a system that enables patients to access health education and support for self-care usually through the internet [32]. In line with Colucci et al [33], wCBT can be seen as a form of telepraxis with applications of interventions, support, and education to the patient by a health care professional. Though there is no consensus definition of telehealth or telemedicine, most definitions include or acknowledge a physical distance between the health care provider and the person receiving health care [34]. In heart failure care, a number of telehealth applications that can be defined as those used for monitoring patients have been evaluated and shown to reduce mortality and heart failure-related hospital admissions compared with usual care. Telehealth applications employing telephone-delivered support have also demonstrated a positive effect in heart failure care [35]. However, the use of telehealth applications for cardiovascular care with asynchronous or text-based communication (similar to most wCBT interventions) appears less common [36,37]. There are a limited number of studies investigating patients’ experiences of wCBT [38]. Previous qualitative studies on wCBT have explored issues related to reducing dropout [39], describing how treatment effect can be sustained over time [40], and investigating the experience of smartphone-based interventions [41]. In heart failure care, evaluations of telehealth applications have mostly focused on applications that are used for monitoring patients [42]. Despite the lack of knowledge regarding how wCBT and other telehealth interventions are experienced by persons receiving health care, there is a drive to implement telehealth applications for the care of persons with chronic somatic disorders [43].

Our research group has recently developed and pilot-tested a 9-week guided wCBT program (Table 1) aimed at decreasing depressive symptoms in persons with heart failure [44]. The length, structure, and way of providing our wCBT program are similar to those of other wCBT programs for depression [45]. The program was developed based on Beck’s description of models of depression [46] and is described in more detail in a proof-of-concept study [47]. However, because previous research has indicated the importance of context adaption of wCBT for persons with chronic somatic diseases [28,29], we chose to adapt the program to fit persons living with heart failure [47]. The results of the study conducted by Lundgren et al [44] were promising because it reported a decrease in depressive symptoms and improvement in health-related quality of life. The study also showed an association between patient activity in the treatment program, age, and sex with the treatment outcome [44]. However, the quantitative design of that study could not provide answers to what aspects were important for activity in the program or how the participants experienced the intervention, underpinning the need for qualitative studies on wCBT. Thus, there is a gap in knowledge regarding how persons with chronic heart failure and depressive symptoms experience wCBT. Exploring the perspective of persons receiving telehealth interventions is important to further develop and improve health care interventions, such as wCBT programs [48,49]. Therefore, the aim of this qualitative study was to explore and describe experiences of participating and receiving health care through a wCBT intervention among persons with heart failure and depressive symptoms.

## Methods

### Design

This was a qualitative, inductive, exploratory, and descriptive interview study [50] using data obtained from participants in a wCBT program.

### Setting

The participants were persons with heart failure and depressive symptoms who were recruited after their participation in a wCBT program for depressive symptoms, hereafter called the wCBT program [44]. The wCBT program consists of 7 modules (Table 1), including text and assignments that the participants work with in their everyday setting. Written feedback was provided on all assignments. The participants could also ask questions through a secure message system. The program did not entail face-to-face or direct interaction (except technical telephone support if needed). A detailed description of the wCBT program is published elsewhere [47].

### Participants: Sampling and Recruitment

To ensure that participants had experience of wCBT, purposive sampling was used [50]. All participants (n=27) that had been active in the wCBT modules during or after the active study were invited via mail to participate in a qualitative research interview. Of those contacted, 13 (9 men and 4 women, aged 41-80 years with median 69, living in Sweden; Table 2) consented to participate and were included in this study. In alignment with the inclusion criteria for the wCBT program, all participants had, at the start of the wCBT program, at least mild depressive symptoms, had been diagnosed with heart failure for more than 6 months, and had not been admitted to hospital for at least one month.

### Data Collection

Semistructured telephone interviews [51,52] using an interview guide (Table 3) were performed between 1-12 months after the program had ended. The participants choose the time and place for the interview.

**Table 1:** Description of the Web-based cognitive behavioral therapy program.

Module and homework assignment		Description of homework assignment given to participants	
**Introduction**			
	Expectations and goal for the program		Describe their expectations and goal for the program
**Living with heart failure**			
	My symptoms of heart failure		Describe their symptoms of heart failure, how much and when these symptoms affected them, as well as what they thought they could do to reduce their problems
	Knowledge about heart failure		Answer multiple-choice questions about heart failure and treatment of heart failure
	How does heart failure affect me?		Identify situations when they were affected by heart failure and suggest changes that might ease the burden of heart failure
**Depression and heart failure**			
	My symptoms of depression		Describe symptoms of depression, how much and when these symptoms affected them, as well as what they thought they could do to reduce their problems
	Knowledge about depression		Answer multiple-choice questions about depression and treatment of depression
	Worries and fears		Identify situations where they felt worries or fear in relation to heart failure, elaborate on what they thought could reduce these feelings, and discuss these issues with a significant other
**Behavior activation 1: To enable change**			
	Activity plan 1		Make an activity plan for one week and assess each activity as positive or negative
	Desirable activities		Make a list of desirable activities
	Increase the likelihood of performing desirable activities		Describe situations that would make it likely that they performed the desirable activities
	Activity plan 2		Implement one or more desirable activities in their activity plan
**Behavioral activation 2: To implement change**			
	Activity plan 3		Review negative activities in their activity plan and reduce the number of negative activities
**Problem solving: A tool for dealing with problems**			
	Practical problem solving		Identify problems in their everyday life, list a number of possible solutions for each problem, test one solution and evaluate the chosen solution
**Consummation**			
	To create an action plan		Review and summarize the program and identify what they have learned to be used in an action plan

**Table 2:** Characteristics of the sample (N=13).

Sociodemographics			n (%)
**Gender**			
	Men	9 (69)	
	Women	4 (31)	
**Family situation**			
	Married or cohabiting	9 (69)	
	Living alone (single, widowed, or divorced)	4 (31)	
**Place of residence (type)^a^**			
	Town with >100,000 inhabitants	4 (31)	
	Urban area with 20,000-49,999 inhabitants	3 (23)	
	Urban area with 1000-19,999 inhabitants	3 (23)	
**Health- and illness-related factors**			
	Mild depressive symptoms at beginning of wCBT^b,c^	7 (54)	
	Moderate depressive symptoms at beginning of wCBT	3 (23)	
	Moderately severe to severe depressive symptoms at beginning of wCBT	3 (23)	
**Completion of** **the wCBT program^d^**			
	1-3 modules	3 (23)	
	4-6 modules	6 (46)	
	7 modules	4 (31)	

^a^Town and urban areas are defined as having at least 200 inhabitants with a maximum distance between the houses of 200 meters information; not available for 3 participants.

^b^wCBT: Web-based cognitive behavioral therapy.

^c^Depressive symptoms assessed with Patient Health Questionnaire-9. The following cut-off scores were used: 5-9 mild depressive symptoms; 10-14 moderate depressive symptoms; ≥15 moderately severe to severe depressive symptoms.

^d^The wCBT program consisted of a total of 7 modules designed to be used during a 9-week period.

The interviews lasted between 36-72 minutes (median 50 minutes) and were performed by JL (9 interviews) and AKK (4 interviews). Both interviewers were registered nurses. JL performed the interviews in his role as a PhD student and AKK in her role as a lecturer. Both had a good contextual understanding of the program. JL had some previous experience and AKK had extensive experience of qualitative interviewing. JL had previous contact (emails and writing feedback) with the participants during the program. All 13 interviews were digitally recorded and transcribed verbatim, 7 by a professional secretary with experience and 6 by JL.

### Data Analysis

**Figure 1:**
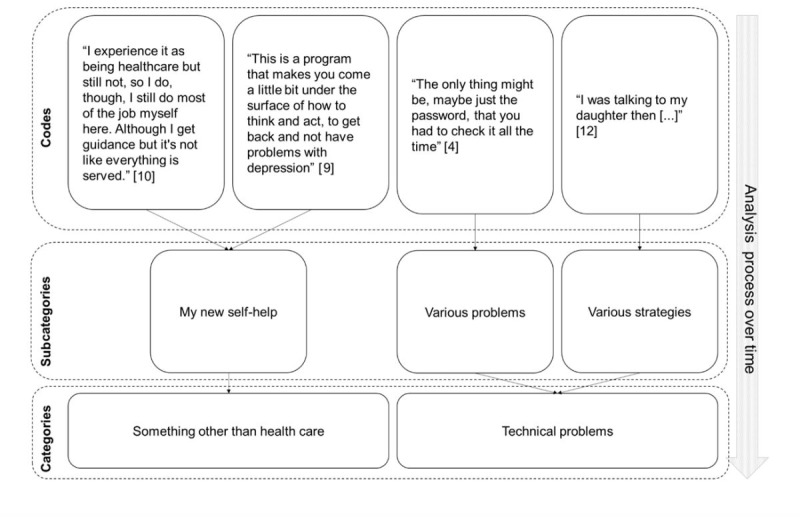
Example of the data analysis process from code to categories. Codes consist of text chunks from the transcripts. Codes with similar meaning were sorted into categories and subcategories. Participant ID shown in brackets.

First, all transcripts were checked for accuracy against the recordings. Second, all transcripts were read as a whole to become familiar with the data. Third, a coding scheme was developed by JL and AKK. JL and AKK read and independently coded one interview. Anonymized parts of 3 transcripts were then used to generate tentative codes during a data workshop with PhD students from the disciplines of nursing and occupational therapy. The tentative codes generated by JL, AKK, and the workshop participants were then discussed by JL and AKK and a refined coding scheme was developed. Based on the developed coding scheme, JL then systematically coded all transcripts. At the end of the coding process, one more transcript was selected and coded by both JL and AKK to ensure a fair and neutral application of the coding scheme [50]. Fourth, codes were sorted into inductively emerging patterns to form subcategories and categories. After all of the codes were preliminarily categorized, the categories were examined to assess whether themes could be constructed [50]. The construction of subcategories, categories, and themes was documented in a memo that also was checked by AKK for clarity and understanding; if necessary, preliminary categories were revised in accordance with the consensual understanding of JL and AKK. Lastly, preliminary categories were either reformulated as final categories or incorporated with other categories with similar content into final categories. The final categories were checked for confirmability [50] by PJ. Figure 1 shows an example of the analysis process.

### Ethics

All participants were informed about the study and gave informed consent. To protect their confidentiality, published data were scrutinized to prevent the identification of the participants. The study conformed to the principles for medical research, as described in the World Medical Association’s Declaration of Helsinki [53], and was approved by the regional ethical review board in Linköping, Sweden (dnr 2011/166-31).

**Table 3:** Interview guide.

Opening question	You have (recently) participated in a Web-based cognitive behavioral therapy program (targeting depressive symptoms in people with heart failure). Can you please tell me about this?
If not spontaneously addressed, ask about the following topics or areas:	Advantages and disadvantages of the treatment program Hindrances and barriers and problems due to the fact that the treatment was given through the internet? How did you cope with this? Positive aspects of treatment through the internet? How would you describe what the program was or is? Contact with the feedback provider Experience of the feedback Use of the internet for any other type of health care? What do you think about the health care system using the internet more for information and interventions? In your experience, what is the difference between health care provided face-to-face compared with health care provided through the internet? What are the advantages and disadvantages? Integrity and health care through the internet.
Probing questions to be used during the interview	Can you tell me more about...? Can you explain or clarify what you mean...? Earlier you said...? Can you give an example...?
Ending	Is there anything more you would like to tell me? Is there anything we have not talked about?

## Results

### Main Findings

Overall, 6 categories were identified during the analysis process. The categories, as well as the included subcategories, are listed in Table 4. One central and common pattern in the findings was that the participants experienced the wCBT program as something they did themselves and many described the program as a form of self-care.

### Something Other Than Usual Health Care

The participants described wCBT as a multifaceted experience. A common pattern was the experience of actively doing something to feel better or gain health. wCBT was described as a good way to treat depressive symptoms. Most participants described their experiences of wCBT in relation to other types of health care interventions they had encountered. Two subcategories were identified.

*My new self-help* meant that wCBT assisted with self-help or self-care that helped participants to actively learn new ways to take responsibility for their health. Some participants stressed that wCBT was about learning and described the program as a course. Other participants said that wCBT could be similar to health care because professional health care personnel were accessible through the program but that wCBT was different from what they generally thought of as health care. However, most participants were hesitant about using words such as “health care.” This was due to their experience of having to be very active. wCBT was experienced as something one did in contrast to health care, which was seen as something one received.

Yes, it is, kind of, a good reason to make, or maybe get help to make these changes that...And maybe I can get some advice that I hadn’t thought of myself...Kind of like someone taking one’s hand and saying: Let’s do it this way.

Participant 10

*Helping other people* meant that the program was experienced as a research project with the purpose of helping people with heart failure. Codes in this subcategory often came from participants describing the wCBT program as a treatment that was not mainly focused on them as individuals but an experience of contributing to other people’s well-being was conveyed.

I think that it’s the right thing to do to give one’s support. If I can contribute in any way to research then I’ll do so willingly.

Participant 3

### Relevance and Recognition

Most participants experienced the program as relevant and useful, at least to some degree. However, a few participants experienced no benefits from the program. An important factor related to finding the program useful was to recognize oneself in the program.

*Mainly useful* meant that some participants experienced the program as highly relevant. A few participants described no experience of any shortcomings in the content of the program. However, most participants generally described the content of the program as helpful but with some disadvantages, such as describing the behavioral activation as hard to grasp or experiencing that the content of the program did not address areas and topics that they thought important for their health, such as management of health problems other than heart failure and stress management.

Then I thought that it [the program] would probably contain a little bit more when it came to tools for stress too [...] There was a little but it didn’t give me those...I would have really liked a pure exercise program [to handle stress] there.

Participant 9

In contrast to *Mainly useful*, in *No use,* a few participants reported that the program had not been of any benefit for them.

No, I don’t really think I got anything that I considered a cure if I can put it like this. [...] No, I might as well say that it was not of any benefit.

Participant 7

**Table 4:** Overview of findings.

Categories	Subcategories
Something other than usual health care	My new self-help Helping other people
Relevance and recognition	Mainly useful No use Different levels of recognition
Flexible, understandable, and safe	Working at home When I want and have time Adapting the content Challenging format and medium Everyday life affecting the treatment Integrity is protected Written format Anonymous and honest
Technical problems	Various problems Various strategies
Improvements by live contact	Depending on the circumstances Preferred situations
Managing my life better	Feedback is confirming and motivating Reflecting and new understanding

*Different levels of recognition* meant that experiences of recognition in and relevance of the program were commonly described by the participants. Examples and problems addressed were found to be realistic and similar to their everyday life. Furthermore, if participants described CBT components in the program as helpful, this contributed to their experiences of the program being recognizable and relevant.

That’s the way it is, and I’m the one who is most positive about the program because it’s worked for me from start to finish.

Participant 8

However, in contrast, some participants did not recognize themselves in the program. The reasons for this were described as feeling too well or too ill compared with the presented examples. Another reason was described as having health problems not addressed in the program.

That is to say some of the examples that you gave there [in the program] didn’t seem relevant to me.

Participant 7

### Flexible, Understandable and Safe

The participants experienced working through the internet as positive and flexible. There were also challenges and some barriers connected to the use of the internet; 8 different subcategories were identified in this category.

*Working at home* meant that most participants positively described working with the program at home because the home environment was peaceful and quiet. Working at home reduced the barriers to participating in a treatment program because one did not have to travel. This was important because getting started with different activities was experienced as difficult when suffering from depressive symptoms.

Let’s put it this way, it was really nice to be able to be at home and do it in peace and quiet.

Participant 4

*When I want and have time* meant that the format and medium of the program provided a positive opportunity to work when the participants wanted to. Feeling motivated was described as important when choosing when to work with the program. In addition, the ability to increase or decrease the work tempo and repeat parts of the program were experienced as positive aspects. Contributing to the flexibility was the opportunity to make adjustments to when and where to work with the program, for example, if participants made a trip.

If I wanted to sit down and do it at two in the night or five in the morning or in the middle of the day then this was fine I could choose when to carry out my exercises [...] it is an advantage to be able to do it at a time of my choice.

Participant 6

*Adapting the content* meant that participants described choosing to focus on the parts of the program that they experienced as meaningful and putting less effort into parts experienced as less important. The parts of the wCBT program that participants chose not to work with concerned things they already knew or did.

I located and picked those small components, now what were they called? Little gems, [...] Oh, they were part of the internet program and they worked for me and were great.

Participant 8

*Challenging format and medium* meant that the format and medium made it easy to postpone the work on the program. Some participants therefore thought of setting fixed times to work on the program despite it reducing the flexibility. The experience of the program requiring self-discipline was common. The work was often experienced as lonely, especially if participants encountered problems with the program. The participants experienced a need to be active and take the initiative to solve some problems by themselves to gain a positive effect from the program.

I’m a bit like, I’ll do this later and this bit I’ll postpone. And this is exactly what happens, and then things get difficult [...] It was not always a good thing that it was Internet-based and things got postponed.

Participant 4

Other challenges described were that the program required a lot of time and that there was a lot of text and assignments that needed to be completed during the program, which was experienced as stressful. In contrast, some participants described the program as timesaving owing to the medium and format. This was because the wCBT program gave the participants the opportunity to focus on the parts they felt important and because they did not have to travel to receive health care.

The participants also said that they were not used to working with treatment through the internet, but they did not clearly assess the medium as negative or positive—rather, most participants described it as unusual. Another aspect experienced as valuable was the possibility to read the content of the program in printouts or on screen. Commonly, participants said that they were not used to reading long texts on the screen. Being presented with a number of different choices on how to perform the tasks in the program was sometimes experienced as hard, especially if the participants were not used to working with information technology and computers. Participants also said that the medium and format made them feel tied to the computer and vulnerable if the technology did not work. In contrast to the challenges experienced above, some participants mentioned no challenges with the medium and format.

*Everyday life affecting the treatment* meant that factors or events not directly related to the program, heart failure, or depressive symptoms were important for how wCBT was experienced. These factors sometimes made it harder to work with the program, for example, if there was a demanding situation at work or if they had to handle other health problems.

Just when one starts to establish a routine, things crop up which get a higher priority, and then these objectives and reviews get put on the back burner.

Participant 9

*Integrity is protected* meant that no experiences were identified of integrity being insufficiently protected in the program. The security system used in the program made them feel safe in regard to how information about them was handled. The organization behind the program was also important for creating experiences of safety. Universities and other public institutions were described as more reliable compared with private companies. Some participants experienced that integrity generally received too much attention when information technology systems were discussed.

Look here, I’m 75 and have a number of medical problems and I don’t give a damn about confidentiality just as long as I receive decent health care.

Participant 2

However, among all the participants there was a limit to how, where, and by whom the information should be accessed; for example, some participants said that they would not be comfortable if such information was spread through social media. The participants said a number of times that it was important that health-related information was accessible for health care personnel, even if this came at the price of unauthorized persons being able to access the information. Close relatives such as spouses were also described as persons one may want to protect personal information from, something that was described as complicated when working at home with the program.

...it is worse if one is writing something that one doesn’t want the family to see...it can be a negative aspect of such a course of treatment if one has an inquisitive partner, indeed then things can get quite difficult.

Participant 8

In *Written format,* the experiences differed in that the program mainly consisted of written information and depended on written communication. Some described the texts and feedback as well thought out and easy to read and understand. Furthermore, it was experienced to be easier to write about some types of health problems compared with talking about them. The written format was described as facilitating and clarifying what the participants were expected to do when working with the program. In contrast, some experiences suggested that the written format was a barrier to communication, and descriptions revealed that oral communication between participant and feedback provider was preferred.

Exactly, one gets a question which one starts to answer [...] one understands exactly what they’re after [...] it’s the way I am [...] it can be difficult to express oneself [verbally].

Participant 4

*Anonymous and honest* meant that working through the internet created conditions for a positive experience of anonymity. The participants thought that they could write exactly what they thought about things and be more honest in their communication. They were also positive about not feeling observed or analyzed. A requisite for this was that the communication took place without personal encounters. Furthermore, written delayed communication led to experiences of the feedback provider as a neutral medium or an unknown person. Both perceived anonymity and written delayed communication contributed to the experience of being protected or doing something behind a screen.

Yes, this I would recommend, namely that one can be completely open there is no need to feel observed or analyzed or monitored in any way–instead it all takes place behind a screen, so to speak.

Participant 6

### Technical Problems

A number of technical problems were reported during participants’ work with the program. However, not everyone described such problems, and most participants experienced that they had been able to manage the problems they came across.

*Various problems* were encountered and experienced in 4 different ways. First, problems were associated with participants’ computer equipment, such as old computers and problems with viruses. Second, the technique used in the program was demanding and complicated. Third, the interface of the program was sometimes confusing, creating insecurity regarding how to perform different tasks; for example, there was lack of automated feedback to know if assignments had been submitted.

[...] when one had submitted something then one was uncertain as to whether it had arrived.

Participant 12

Fourth, technical problems during the log-in and authorization process were described. The log-in process could be complicated and some stated that they would have preferred to be able to set a less complicated password. *Various strategies* meant that technical problems were managed in different ways. One strategy was to postpone the work if a technical problem was encountered, assuming that the problem would have been solved the next time one tried doing a task. Support was sought from relatives, friends, or the feedback provider. One participant said that she chose to get a new computer of the same model as a relative to get support that way. Another strategy was to read the instructions and test different solutions on one´s own.

Yes, I had to wait, repeat and try again. Yes, now and then I contacted [name of feedback provider] for assistance. [...] I sat there and explored the software by myself.

Participant 10

### Improvements by Real-Time Contact

Most participants were less used to indirect contact with health care personnel. This was sometimes experienced as challenging and demanding. Participants described this as having to learn a new way of communicating with health care personnel. Several participants stated that they would have preferred more real-time contact, that is, direct verbal communication via telephone or video-telephony. Real-time communication through sound and picture (not necessarily only face-to-face) was described to make communication more dynamic and was thought to facilitate relationship building better than indirect communication. These experiences were described regardless of whether participants described the feedback in the program as positive and supportive or not.

*Depending on the circumstances* meant that the experiences of the need for real-time communication were described as more important in some situations and less important in other situations. The purpose of an intervention or interaction was crucial for how important real-time communication was; for example, real-time communication was important for feeling cared for. Some participants said that delayed and indirect contact made them feel anonymous or reduced to a number. In contrast, when the experience of the program was described to be about learning, which was common, the delayed and indirect contact was experienced as suitable and sufficient.

Oh yes, the obvious way is a face-to-face meeting with a person. But the thing with CBT and the like, and learning generally...Obviously things go really well with the Internet perhaps it is even an advantage.

Participant 11

*Preferred situations* meant that more real-time contact would have been preferred to allow for check-ups of the progress in the work and how the participant was doing. The use of real-time communication as a back-up if something did not work was also stressed.

For example suppose that you gave feedback via phone in a different way. Then one can explain a bit more and other things too.

Participant 5

### Managing My Life Better

The participants described that they had generally become better at managing their lives. This was expressed in different ways among the participants. Some participants had learned to stop activities perceived as negative and had started to do things that balanced their activities in daily life. Others said that they had started to take the initiative to perform more positive activities and that they had begun to appreciate life again.

The participants experienced that problems could be solved and tasks could be performed differently compared with before they participated in the program. A prominent pattern was the experience of the program as stimulating them to take a greater responsibility for their health, for example, by seeking information about health problems and discussing health problems with their close relatives. This behavior change made them feel safer, reduced their feelings of hopelessness, and helped them to gain greater acceptance of their own health problems. The following 2 subcategories were identified: *Feedback is confirming and motivating* and *Reflection and new understanding*.

*Feedback is confirming and motivating* meant that the feedback participants received from the feedback provider was important because it facilitated the work process. One divergent finding was that a few participants described the feedback as not powerful enough to create a change in behavior. However, the feedback mostly was described as good and sometimes as prompt.

But no, I think it would give a little more I mean you need some pressure, you need a carrot and stick you know.

Participant 2

The feedback was described as answering questions and providing new perspectives on the health problems that the participants worked with. Participants said that the feedback helped them to continue working in the program even if it felt cumbersome at times. Adding to this, the following 3 different experiences of good feedback were identified: confirmatory, coaching, and motivating.

And receive an answer, an answer of concern to me alone. One doesn’t get standard answers.

Participant 2

*Reflection and new understanding* meant that the program gave rise to increased reflection, leading to new understanding and perspectives on their health and life situation. Descriptions showed that it could be painful to accept that heart failure comes with a poor long-term prognosis. On the other hand, experiences of being more prepared to manage worsening in heart failure were expressed. Participants described it as important that they had understood that depressive symptoms could be associated with heart failure and that it was common among patients with heart failure to experience depressive symptoms. Furthermore, participants said that the program had contributed to the experience of having gained new tools to manage problems and it had thereby affected their situation and well-being. They had learned to think in new ways and the program gave them new perspectives on everyday life. A common pattern was that participants started to challenge their old inward thinking. New thoughts were mostly positively expressed, including metaphors such as “the program was an eye opener” or “I see things from new angles.” However, one participant said that the program made him identify his health problems but that he could not take advantage of the program. Instead, he had sought other types of health care.

However, I noted the following: well, I never, that was interesting. One hadn’t seen the connection and context of the two things, but when one got these two questions the penny dropped and one started to think in new ways.

Participant 1

## Discussion

### Principal Findings

The main finding of this study was that persons with depressive symptoms and heart failure described wCBT as a form of self-care for their health problems. Overall, the program was experienced as a new way to create self-care by active reflection on relevant and recognizable situations and support from a trustworthy person that was confirmative and motivating. The program was described as safe without any major challenges to integrity, except from their partners.

The primary target of the wCBT intervention was to decrease depressive symptoms. However, the participants’ narratives most often referred to the program as something they experienced to help them take care of their own health problems, including both physical and psychological health problems. We interpret this as a holistic perspective on health among the participants. It is important to realize that the findings of this study are what the participants experienced and described and not primarily a qualitative evaluation of the wCBT program’s effect on depressive symptoms.

Participants experienced that wCBT required them to be active participants in the treatment process and that the program helped them to focus on what they could do to improve their health. This is consistent with meta-analyses reporting data in studies of cancer survivors [54] and in the field of depression and anxiety [30]. If patients’ and health care professionals’ expectations differ with regard to patients’ active participation, this may be a substantial barrier for telehealth interventions [48]. Consistent expectations regarding the roles and activity between patient and health care provider are an important factor affecting adherence to treatment and patients´ perceived quality of care [55]. Our finding that the participants experienced wCBT as self-care is important and may contribute to a common and good starting point for this type of intervention. The experience of wCBT as requiring the patient to be active and perform self-care with the description of wCBT as a learning process indicates that this type of intervention may not be suitable for all patients. Self-care ability can be negatively affected by learning problems [56]; thus, some patients may need other types of support with their health problems, such as face-to-face interventions. In our findings, the participants described wCBT as a good way of addressing depressive symptoms. This is important because participants’ attitudes to psychological Web-based interventions have been found to be an important factor for response to treatment, as demonstrated by Lutz et al [57]. From a professional perspective, it is important to make a comprehensive assessment of self-care and learning ability as well as the attitudes to Web-based interventions of patients before considering what type of intervention to offer to individual patients.

The above-mentioned findings can be said to have general implications for health care interventions, regardless of how they are delivered. In our findings, there are also some aspects specifically related to wCBT as a telehealth intervention.

Telehealth interventions are often seen as a practical and cost-effective way to face increased demands on a health care system [58]. A wide variety of telehealth applications have been developed, ranging from monitoring health status to the provision of health education and psychological support [59]. A common feature of telehealth interventions is that they change the context in which health care is provided, generally from within health care facilities to the home of the health care recipient. This transition raises questions about personal integrity. The experiences regarding protection of personal integrity were somewhat surprising in our study. Based on the contemporary discourse regarding personal integrity in the context of telehealth (cf. Hall and McGraw [60] or Sabin and Skimming [61]), it was assumed that parts of the interviews addressing this topic would focus on areas such as protection from hackers, nonauthorized third party access, and other more general information technology security topics. Instead, participants described other aspects of integrity as important, such as the need to protect information from close relatives. Moving health care from a traditional face-to-face context to a telehealth context will also move the responsibility for protection and safeguarding of integrity from the health care professional to the patient. Technical solutions, such as passwords, used by the patient can be useful to some extent [62], but these methods may cause other types of problems; for example, passwords on computers, which are often shared among family members, and changing or hiding passwords may be perceived as deception and cause feelings of suspicion, distrust, and dishonesty in the family [63,64].This may have a negative impact on the health process and family function. Therefore, the introduction of telehealth calls for the development of new methods for health care personnel to safeguard their patients’ integrity.

The findings described in the subcategories *Anonymous and honest* and *Written format* with the category *Improvements by real-time contact* reveal a complex relationship between the possibility of being anonymous and a wish to have real-time contact. Anonymity is experienced as an easy way to address sensitive topics and to be honest but at the same time, it also led to an experience of being reduced to a number. Moreover, both positive and negative experiences regarding the use of written material and feedback and the need for more real-time communication were described in our findings. The contradicting findings regarding the written format and real-time communication can be seen as illustrating a dialectic relationship, similar to that described by Knowles et al [30], with participants acknowledging and appreciating the benefits of perceived anonymity and written format but at the same time, longing for the benefits of real-time contact. Acknowledging that this relationship exists in telehealth interventions, such as wCBT, raises a number of questions related to the way health care personnel can act to establish stable and caring relationships and to determine the right amount of personal contact between patients and health care personnel to facilitate optimal care in telehealth interventions. As pointed out by Nagel et al [65], the nursing profession is facing philosophical and transformative challenges as health care increases its use of telehealth. The answers to these questions are beyond the scope of this study; however, this study may contribute to future research that aims to investigate how a caring relationship can be established in a telehealth context. Such research is needed to prepare the present and future generations of health care professionals to deliver high-quality health care when using telehealth applications.

### Methodological Limitations and Considerations of the Study

There are several limitations to this study. The authors’ involvement in designing the program may have affected their preunderstanding in regard to the analysis and presentation of findings [50]. Because the findings represent a construction made by the authors, it is most likely that the findings would be different if the data were analyzed by other researchers. In addition, another sample would most likely render a different finding. In qualitative content analysis, this is a phenomenon that the reader must be aware of [50]. The fact that different findings can be constructed from the same data does not mean that one of these findings is more or less true compared with another construction; instead, they give different perspectives of the phenomenon studied. To enable the reader to assess the trustworthiness of our findings, we have described the setting (Table 1). To further increase trustworthiness, we have provided a detailed and transparent description of the analysis process [66]. To create a credible construction of the findings [50], multiple analysts’ triangulations were used. We also considered member checking [50]. However, it has been proposed that this may lead to confusion about the findings rather than confirmation [67]. Reflexivity and dependability were facilitated by memo writing and by reporting deviating findings of the particularities of the analyzed cases that have been brought out [50].

The use of 2 different interviewers was important to counterbalance the risk of informant or researcher bias. No considerable differences were detected between the interviews conducted by JL and those conducted by AKK.

We performed 13 interviews, which may be considered a small number. However, there are no clear guidelines for an appropriate sample size in qualitative descriptive studies [68]. Because no new codes were identified in the coding of the last transcripts, we believe that the data in this study give a broad description of the participants’ experiences of wCBT. Regarding the heterogeneity of the sample [68], the sample may be considered rather homogeneous, which may limit the contexts to which the findings may be transferred. However, because all participants had similar health problems and participated in the same program, the sample gives a rather exhaustive description of the group’s experiences of the program. Regarding transferability, the participants in the sample in this study are younger than the average person living with heart failure. This may limit the transferability of the findings to the general heart failure population.

Interviews conducted via telephone may have rendered less rich data than those conducted face-to-face. However, face-to-face interviews were assessed as less beneficial because they required restrictions in the sampling process given the geographical spread of the participants. The median interview time was 50 minutes, which is similar to that often seen in other qualitative studies using face-to-face interviews [69]. Despite these limitations, we believe that this study contributes important insights into the novel area of patients’ experiences of participating in a wCBT program.

### Conclusion

Persons with heart failure and depressive symptoms experienced the wCBT intervention as challenging. This was because of participants balancing the urge for real-time contact with perceived anonymity, not postponing the work with the program, and learning a new way of communicating with health care personnel. Important advantages of the program were learning by reflection to gain new understanding of health problems and managing self-care to improve health. Being able to choose the time and place for the work was another advantage. wCBT appears to be experienced by persons with heart failure as a valuable tool to manage depressive symptoms. However, more research is needed to identify the circumstances in which anonymity is beneficial for the patients and how wCBT programs should be designed to balance the challenges experienced by persons receiving health care through wCBT.

## Abbreviations

ACC/AHA: American College of Cardiology/American Heart Association
CBT: cognitive behavioral therapy
ESC: European Society of Cardiology
wCBT: Web-based cognitive behavioral therapy
